# Technical Guidance for Clinicians Interested in Partnering With Engineers in Mobile Health Development and Evaluation

**DOI:** 10.2196/14124

**Published:** 2019-05-15

**Authors:** Lochan M Shah, William E Yang, Ryan C Demo, Matthias A Lee, Daniel Weng, Rongzi Shan, Shannon Wongvibulsin, Erin M Spaulding, Francoise A Marvel, Seth S Martin

**Affiliations:** 1 Johns Hopkins University School of Medicine Baltimore, MD United States; 2 Johns Hopkins University Whiting School of Engineering Baltimore, MD United States; 3 David Geffen School of Medicine at University of California, Los Angeles Los Angeles, CA United States; 4 Johns Hopkins University School of Nursing Baltimore, MD United States

**Keywords:** mHealth, cardiology, myocardial infarction, personalized medicine

## Abstract

The explosion of mobile health (mHealth) interventions has prompted significant investment and exploration that has extended past industry into academia. Although research in this space is emerging, it focuses on the clinical and population level impact across different populations. To realize the full potential of mHealth, an intimate understanding of how mHealth is being used by patients and potential differences in usage between various demographic groups must also be prioritized. In this viewpoint, we use our experiences in building an mHealth intervention that incorporates an iOS app, Bluetooth-enabled blood pressure cuff, and Apple Watch to share knowledge on (1) how user interaction data can be tracked in the context of health care privacy laws, (2) what is required for effective, nuanced communication between clinicians and engineers to design mHealth interventions that are patient-centered and have high clinical impact, and (3) how to handle and set up a process to handle user interaction data efficiently.

## Introduction

Mobile health (mHealth) interventions promise to improve patient outcomes, increase self-management and health literacy, reduce health disparities, increase access to health services, and lower health care costs in ways previously unachievable [1,2]. However, much work is needed to move mHealth from the ivory tower of academia or the offices of startup companies to clinical practice, where mHealth interventions can be prescribed as adjuncts to therapy. To facilitate clinician and policy-maker decision making, researchers need to create an actionable knowledge base to identify the most effective, safe, and scalable interventions for improving individual and population health.

mHealth studies evaluating clinical and population-level impact have made strides in this direction. For example, BlueStar by WellDoc, a Food and Drug Administration (FDA) cleared mHealth intervention for patients with type 2 diabetes, demonstrated a significantly lower mean decrease in hemoglobin A_1c_ over 1 year compared with the usual care group [3]. Similarly, a 2016 systematic review and meta-analysis found that across 27 studies, the mHealth group compared with the usual care group had increased adherence to medical therapy and ability to reach blood pressure and exercise goals [1]. However, other studies have found mixed results from mHealth interventions and have further concluded that the evidence for efficacy is still limited [4]. One concern that has been raised is the diversity of patient demographics and how this might lead to differential intervention usage and effectiveness. Indeed, studies have found that sex, age group, ethnicity, and family history significantly affect user engagement and that patients who are more engaged with a given mHealth intervention may be more likely to achieve the intended clinical outcomes [5-7].

Thus, to realize the full potential of mHealth interventions, validation of individual clinical outcomes is not enough. mHealth interventions are typically complex with multiple aims and components, including educational material, self-monitoring tools, and various other aspects of health behavior change constructs. Making meaningful insights and developing recommendations require understanding the causal model of how the intervention will achieve its intended benefit, how key components of the intervention interact with one another, and what combinations will optimize a return on investment [8]. Smartphones, in addition to functioning as a platform for such interventions, send data to a back-end system, where it can be leveraged to understand intervention use patterns in a way that avoids potential limitations of self-reported survey data such as recall bias. This kind of granular app usage analysis, while traditionally not associated with clinical research, is necessary for transitioning mHealth interventions toward real-world implementation and answering the question, “What works and for whom?”

Importantly, an analysis of mHealth back-end data presents important challenges, and few studies have examined clinical outcomes in the context of app usage data [8]. Here, we use our experiences in building the Corrie mHealth intervention as a framework to explore key questions that arise in constructing meaningful insights from back-end data. Corrie guides patients through recovery after acute myocardial infarction and incorporates an iOS app, a Bluetooth-enabled blood pressure cuff, and an Apple Watch. In addition to sharing nuances identified from our research and development process, we aimed to provide practical knowledge to help other clinicians work effectively with an engineering team to design mHealth interventions that are patient-centered and have high clinical impact.

## How is User Interaction Data Tracked?

The back-end data analysis allows mHealth teams to identify features that patients use most regularly and take that into account in an iterative design process, thus quantifying and supporting insights collected through patient surveys and focus groups. This enables teams to begin to understand if specific features may be associated with improved clinical outcomes and ultimately helps make interventions more patient-centered.

Although there are a number of different ways to track app usage [9], our intervention tracking is divided into 2 main categories: (1) the tracking of unique *events* and (2) the recording of time elapsed on *view controllers*. Such collection of intrasession measures (within sessions) of user engagement allow for the evaluation of specific user interactions and behaviors, while intersession measures (across sessions) evaluate long-term user engagement and satisfaction [9].

**Figure 1:**
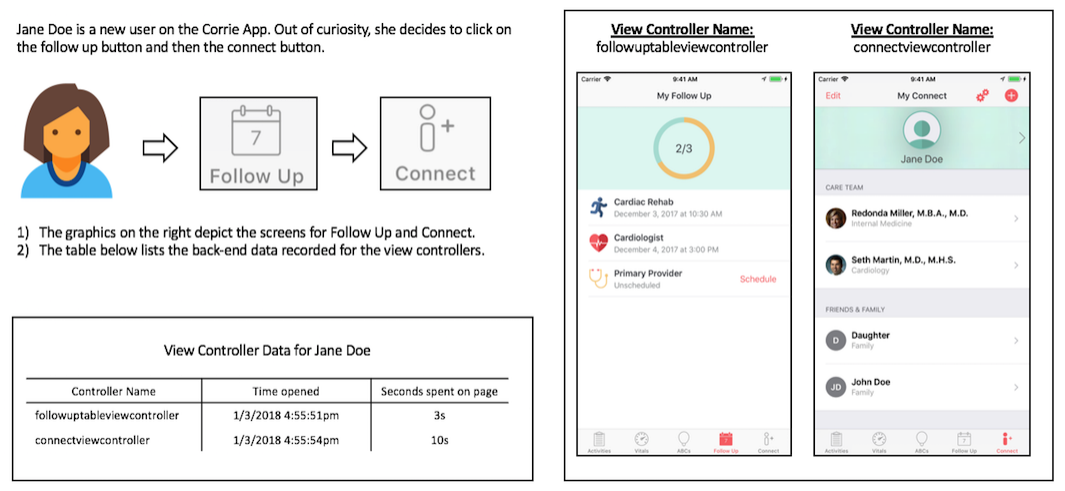
An example of view controllers and their use in user interaction tracking.

In the Corrie iOS app, unique app *events* are generated any time a patient (user) undertakes a specific action—for example, taking vitals, creating follow-up appointments, and completing learning goals. *View controllers* correspond to individual screens on iPhone. These *view controllers* can then be grouped by the feature they are a part of, allowing for the identification of features that patients spend the most time on (Figure 1). Commonly, nonhealth apps outsource this kind of analytics to third-party software development kits (SDKs) such as Firebase and Google Analytics. However, as many of those third-party software tools are not Health Insurance Portability and Accountability Act (HIPAA) compliant, mHealth teams may need to create their own HIPAA-compliant implementations, especially for detailed insights that can be correlated to patients and their clinical outcomes [10].

To make such meaningful insights, clinical teams must have continuous back-and-forth discussions with their engineering team to understand the context of data being obtained and the nuances of how it is captured. In addition to improving team efficiency, this detailed, continuous communication minimizes misinterpretation of the underlying data that could ultimately yield false conclusions. However, achieving this level of communication can be complicated by a technological language barrier and the inherent intricacies of back-end data collection.

## What Do Clinicians and Engineers Need to Know About the Other Side?

**Figure 2:**
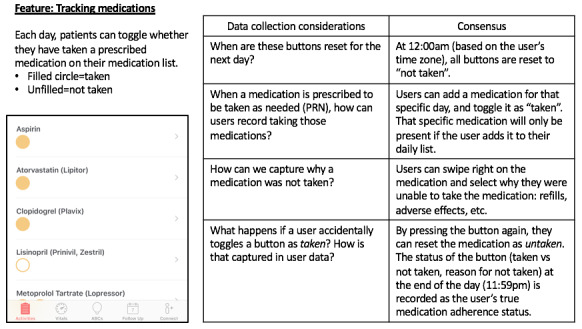
An example of data collection and product considerations when developing a new feature.

To address this, we found that it was critical for the clinical and engineering teams to collaborate more closely than is traditionally done. For example, our clinical team obtained the list of *view controllers* from our engineering team and classified them into 4 broad categories for feature usage analytics. We also walked through multiple use case scenarios with our engineering team to understand and fine-tune how data would be recorded in various clinical situations. This was particularly important for the development of a robust system to track unique app *events*. For example, we wanted to capture both time spent watching an educational video as well as completion of the video. If a patient went back to watch a video multiple times, we also wanted to capture that. However, if a patient accidentally pressed a *medication taken* button multiple times, we wanted that recorded as one *event* rather than multiple *events* (Figure 2).

Beyond recording app usage *events*, we also wanted to capture the values of clinical measurements such as heart rate, blood pressure, step count, weight, and mood to create rich datasets for a health outcome–based analysis. For these types of data, we emphasized the importance of querying and displaying the frequency of measurement and timestamps, as well as having well-defined data collection time windows. These details require a significant understanding of variable-capturing capabilities on the back-end but we believe they are key to building accurate and clinically relevant datasets. Ultimately, ensuring high quality and appropriately contextualized data will be critical as interest grows in applying machine learning–based analytical tools to generate actionable insights from large volumes of mHealth data, in the setting of both mHealth validation research as well as clinical scenarios using patient-generated data from commercial devices.

Similarly, when clinicians and engineers team up to solve a problem and work together as equal partners, the engineering team can gain an in-depth understanding of the clinical picture over time. Initially, many mHealth interventions start off with ad hoc combinations of the engineering team and interested clinical professionals partly serving as the data scientists [11]. For our team, this meant that when data were needed, the engineering team provided the relevant information to the clinical team. Close collaboration with the engineering team was required to sort through all the recorded variables and determine which were important to analyze and which were not. To facilitate that decision making, the engineering team needed to have a strong understanding of the clinical context. In addition to taking our engineering team through the discharge process and hosting our engineers on rounds in the hospital, we found it helpful to come together with our engineering team in clinically focused discussions by using real-time team messaging and collaboration platforms such as Slack (Slack Technologies, Inc). We have found this more efficient and effective than email and it provides an ongoing, continuous form of discussion outside the context of our regular face-to-face meetings and calls to facilitate clinical engineering collaboration. Slack discussions enable moment-to-moment understanding of clinical challenges, with the bonus of being able to answer urgent technical questions in real time. Importantly, this streamlined troubleshooting and prioritization of tasks for our engineering team as well, who were able to leverage real-time clinical perspective on potential features, content, and user workflow.

## Who Handles User Interaction Data and How Is This Affected by Health Care Privacy Laws?

Teams developing mHealth interventions must recognize that having the engineering team manually export back-end data is not a feasible long-term solution. As the intervention scales and the clinical team’s need for data extraction increases, the potential for severe bottlenecks warrants an early discussion about data management and access. For example, as our clinical team expanded, it became clear that identifying appropriate variables and packaging data into easy-to-read formats cost the engineering team valuable time that could have been better spent on other endeavors such as further developing the app.

**Figure 3:**
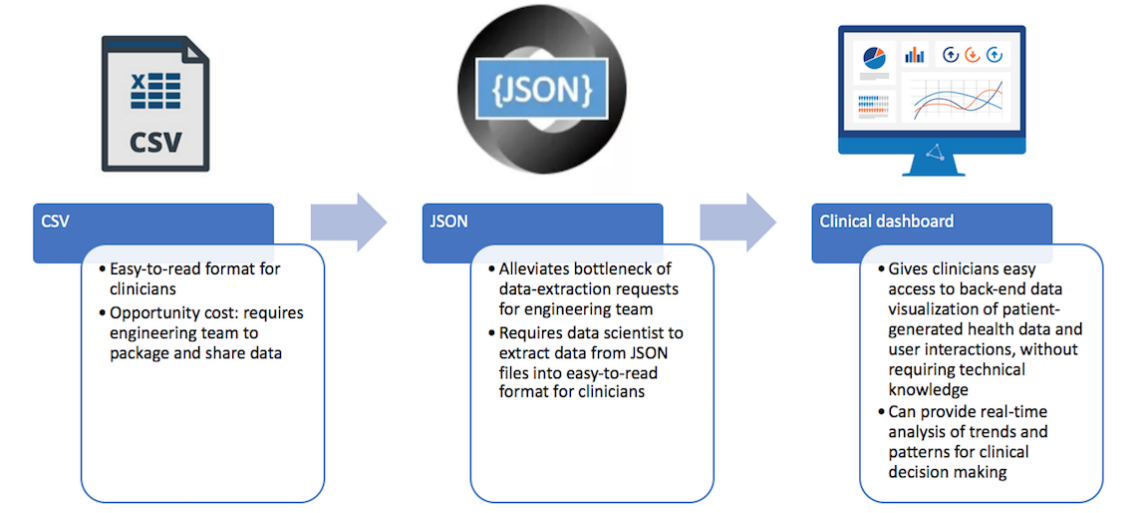
Progression of user-interaction data management and extraction. CSV: comma separated value; JSON: JavaScript Object Notation.

In our case, this led to a stepwise progression of methods to access back-end data in a HIPAA-compliant manner. At first, our engineering team shared data via standard comma separated value (CSV) files over the HIPAA-compliant Johns Hopkins secure virtual desktop. We then transitioned to sharing a JavaScript Object Notation (JSON) file (a data-interchange format) and hiring a data scientist, giving clinicians access to all of the back-end data without the need for extensive teamwide technical training. Although we advocate hiring a dedicated data scientist to bridge the gap between clinicians and engineers early on, as studies scale up and move toward pragmatic trials that evaluate real-world implementation, we believe the end-goal also includes an HIPAA-compliant, queryable dashboard for clinicians, with data visualization and electronic medical record integration (Figure 3) Creating a system that allows clinical team members to intuitively access raw usage data not only decreases the burden on engineers but also empowers team members to better understand their data architecture and more confidently perform analyses based on their individual priorities.

## Conclusions

**Figure 4:**
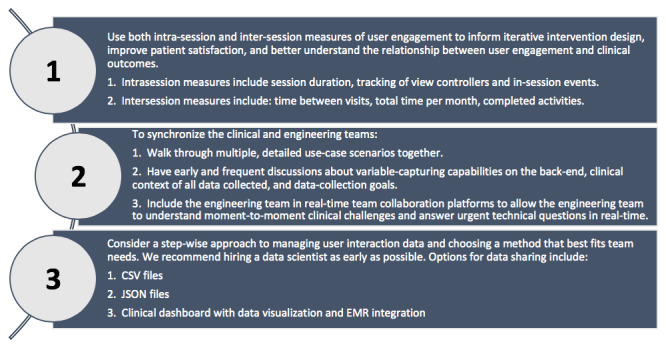
Key takeaways. CSV: comma separated value; JSON: JavaScript Object Notation; EMR: electronic medical record.

Going forward, it will be essential for research teams to evaluate mHealth interventions in the context of potentially differential app usage patterns and to create environments that support continuous, technically nuanced communication between their clinical and engineering counterparts. Although more business than clinical in nature, these changes to practice are essential to building interventions that patients actively want to use in real-world settings. In our experience with mHealth, being consumer-centered is synonymous with being patient-centered and is the future of health care delivery. We hope our experiences help interdisciplinary teams navigate similar challenges (Figure 4), as the capacity of mHealth interventions to revolutionize health care is directly linked to the quality of interventions developed through clinical-engineering partnership and studied through careful research.

## Abbreviations

CSV: comma separated value
FDA: Food and Drug Administration
HIPAA: Health Insurance Portability and Accountability Act
JSON: JavaScript Object Notation
mHealth: mobile health
SDK: software development kit
