# Correction: Technical Guidance for Clinicians Interested in Partnering With Engineers in Mobile Health Development and Evaluation

**DOI:** 10.2196/41813

**Published:** 2022-08-18

**Authors:** Lochan M Shah, William E Yang, Ryan C Demo, Matthias A Lee, Daniel Weng, Rongzi Shan, Shannon Wongvibulsin, Erin M Spaulding, Francoise A Marvel, Seth S Martin

**Affiliations:** 1 Johns Hopkins University School of Medicine Baltimore, MD United States; 2 Johns Hopkins University Whiting School of Engineering Baltimore, MD United States; 3 David Geffen School of Medicine at University of California, Los Angeles Los Angeles, CA United States; 4 Johns Hopkins University School of Nursing Baltimore, MD United States

In “Technical Guidance for Clinicians Interested in Partnering With Engineers in Mobile Health Development and Evaluation” (JMIR Mhealth Uhealth 2019;7(5):e14124) the authors made two updates.

The corresponding author’s telephone number was changed to 1 410 550 3350, and their email address was changed to lochanshah2019@gmail.com.

The corrections will appear in the online version of the paper on the JMIR Publications website on August 18, 2022, together with the publication of this correction notice. Because this was made after submission to PubMed, PubMed Central, and other full-text repositories, the corrected article has also been resubmitted to those repositories.

